# Heterogeneous impact of Covid-19 response on tuberculosis burden by age group

**DOI:** 10.1038/s41598-022-18135-6

**Published:** 2022-08-12

**Authors:** Boyeon Kim, Young Ae Kang, Jeehyun Lee

**Affiliations:** 1 School of Mathematics and Computing (Mathematics), Yonsei University, Seoul, Republic of Korea; 2 Division of Pulmonary and Critical Care Medicine, Department of Internal Medicine, Severance Hospital, Yonsei University College of Medicine, Seoul, Republic of Korea; 3 Institute of Immunology and Immunological Disease, Yonsei University College of Medicine, Seoul, Republic of Korea

**Keywords:** Diseases, Mathematics and computing

## Abstract

Apart from the incidence and mortality caused by it, Coronavirus disease (COVID-19) has had a significant impact on other diseases. This study aimed to estimate the influences of COVID-19 pandemic on the incidence of tuberculosis (TB) and the number of TB-associated deaths in Republic of Korea. A dynamic compartment model incorporating age-structure was developed for studying TB transmission and progression using the Korean population data. After calibration with notification of incidence data from South Korea, the TB burden over 6 years (2020–2025) was predicted under the nine different scenarios. Under the scenario of strong social distancing and low-level health service disruption, new TB cases were reduced by 761 after 1 year in comparison to the baseline. However, in the elderly population, social distancing had little impact on TB incidence. On the other hand, the number of TB-related deaths mainly depends on the level of health service disruption for TB care. It was predicted that with a high degree of health service disruption, the number of TB-related deaths would increase up to 155 in 1 year and 80 percent of the TB-related deaths would be in the elderly population. The decrease of tuberculosis incidence is significantly affected by social distancing, which is owing to reduction of contacts. The impact of health service disruption is dominant on TB-related deaths, which occurs mainly in the elderly. It suggests that it is important to monitor TB-related deaths by COVID-19 because the TB burden of the elderly is high in the Republic of Korea.

## Introduction

After the first case of coronavirus disease (COVID-19) in November 2019, there have been 247,000,000 cases of COVID-19 and 5,000,000 COVID-19-related deaths recorded as of conducting this study^1^. In addition to the morbidity and mortality caused by COVID-19, it also has had a significant impact on other diseases^2,3^. The strong lock down, social distancing, and reallocation of health-system resources implemented for controlling COVID-19 have seriously affected other diseases. Infectious diseases including cholera^4^, measles^5^ and dengue fever^6,7^ have emerged as double burden on the healthcare system in resource limited countries. There is a particular concern regarding tuberculosis (TB), one of infectious disease-related mortality, especially in the low- and middle-income countries. Several mathematical modelling studies have predicted^8–10^ the impact of COVID-19 pandemic on TB incidence and mortality in resource-limited countries with a high TB burden. Based on these studies, the numbers are expected to increase by around 5–15% over the period of next 5 years. In addition, there have been reports of increasing diagnostic delay and decreasing reports of TB cases during the year 2020 from several countries^11–15^. Underdeveloped healthcare system in most low- and middle- income countries enfaces shortage of healthcare workers and hospitals, which has resulted in insufficient diagnostic capacity and disruption of continued care for TB^16,17^.

However, the effects of the global pandemic and control strategy of COVID-19 on TB can manifest from various directions. The impact of social distancing, lock down, and health system disruption on TB depends on different COVID-19 situations and TB epidemiology per country. Social distancing and lock down reduce the spread of *Mycobacterium tuberculosis* in the community but also has a negative effect of increasing transmission within the family^18^. Further, the impact of health system disruption may differ by age, socio-economic status, and region in a country^15^.

Among high-income countries, South Korea has a considerable TB burden, even though the incidence of TB has been reported to decline. The burden of TB in the elderly is a challenging problem in South Korea, accounting for more than 45% of the newly diagnosed cases among them in the year 2019^19^. To control the COVID-19 situation in 2020, social distancing and wearing a facial mask without enforcing strong lock downs were the main control strategies implemented in South Korea. The health system was disrupted and there was a decrease in the number of TB cases reported during the COVID-19 outbreak^13^.

We expect COVID-19 to affect TB outcomes differently by age since the burden of TB and COVID-19 is higher in the elderly as compared to the young population. We developed an age-structured mathematical model of TB to evaluate the impact of COVID-19 on TB incidence and mortality in South Korea, one of the high-income countries, especially in the older populations.

## Methods

### Care cascade for active TB and study design

**Figure 1:**
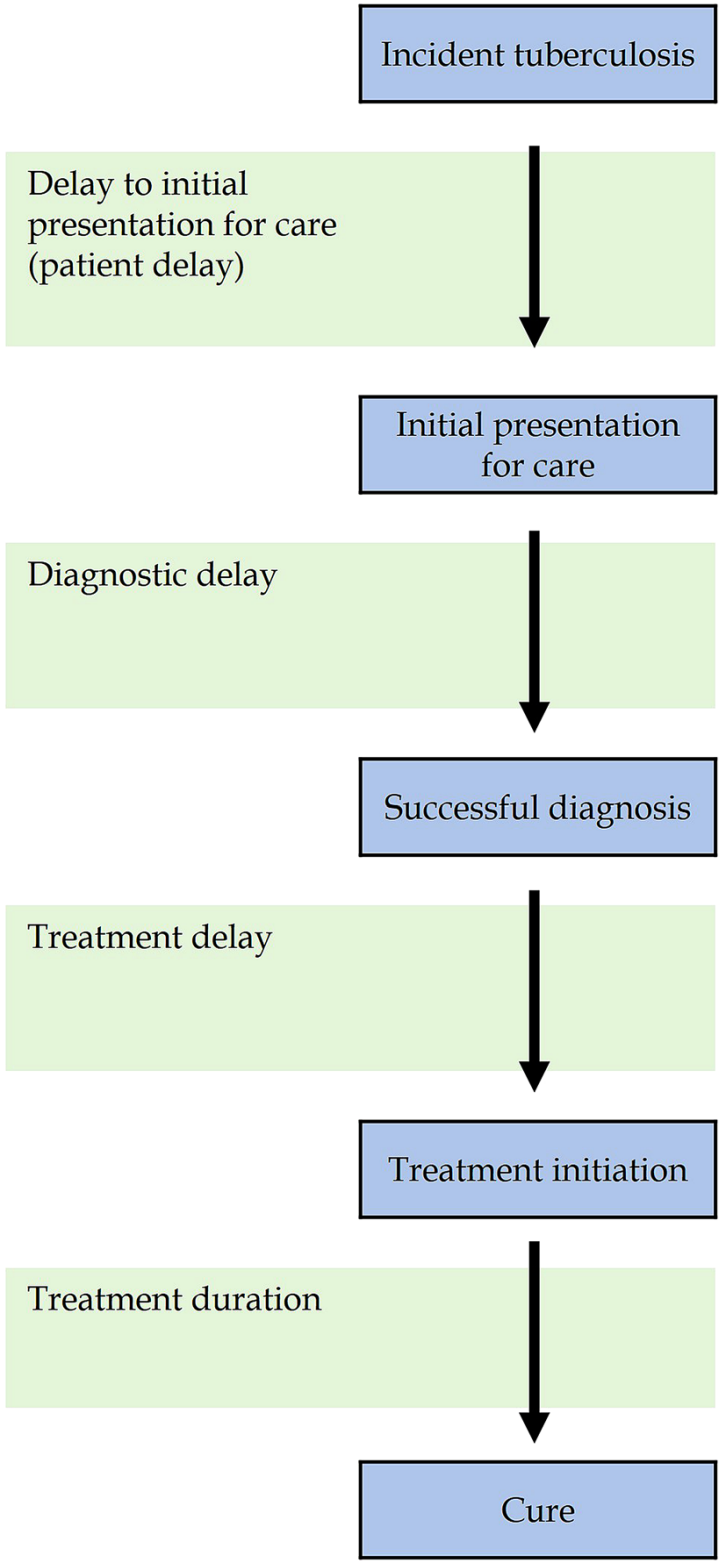
Care cascade and delays for TB treatment^20^.

Several steps are to be followed effective management of TB (Fig. 1)^20^. Patients are successfully treated when all steps are completed. However, we noted that delays might occur at each step for TB treatment, which could affect TB-related incidence and deaths.

Assuming that there would be delays in management of TB due to COVID-19, we developed a deterministic compartment model of TB transmission dynamics incorporating age structure to assess the impact of COVID-19 on TB burden. Under the nine scenarios considering the TB treatment system and the policies in response to COVID-19, the expected number of TB cases and deaths by 2025 are predicted.

### Mathematical model

**Figure 2:**
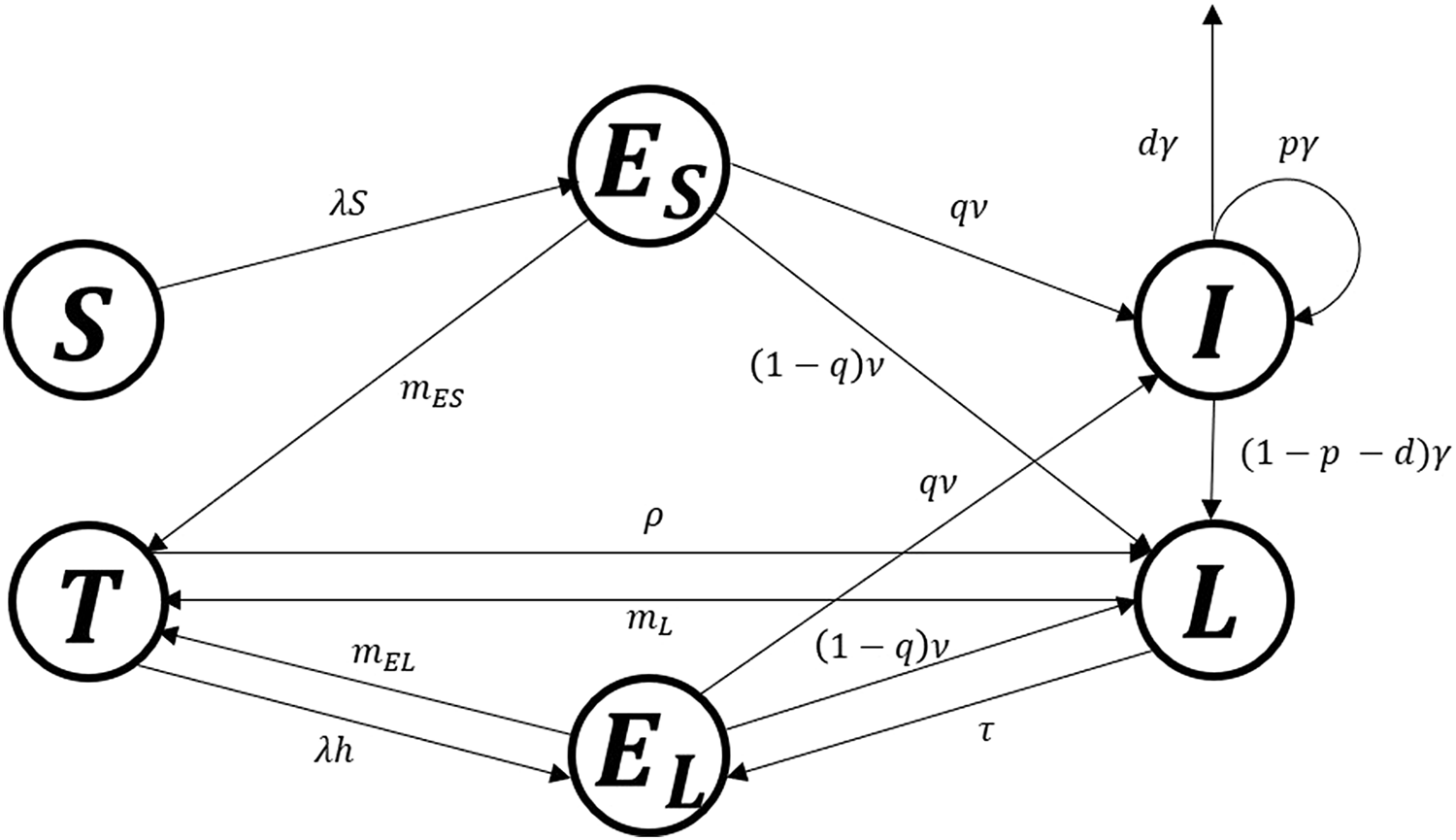
Flow diagram for the TB transmission model.

The Susceptible-Exposed-Infectious-Latent (SEIL) model was modified by incorporating the recently infected group and at-risk-of relapse, reinfection, or reactivation group. The treatment group was also added to show the effect of close contact and latent TB treatment. Figure 2 shows a flow diagram and the system of differential equations for the TB transmission dynamics model. In the SEIL model, the population was classified into six groups based on the disease states: susceptible ($S$), recently infected (${E}_{S}$), at-risk-of relapse, reinfection, or reactivation (${E}_{L}$), infectious ($I$), long term latent ($L$), and treated among close contact and latent TB ($T$). Patients were assumed to be non-infectious during active TB treatment, and successful treatment gave people a similar level of relapse, reinfection, and reactivation risk to patients with long-term latent infections.

To reflect the age-dependent characteristics of tuberculosis in Korea ^21^, each compartment of the SEILT model was further divided by 16 age groups. The main purpose of classifying each compartment into 16 age groups is to reflect the age-dependent characteristics of tuberculosis in Korea. In this study, we considered a model that explained these characteristics incorporating age-specific parameter values such as rate of reactivation, reinfection, or relapse, mortality rates, and preventive therapy. The dynamic structure of the population including birth and death was incorporated using the demographic information of Korea. Next, the age-structured model, integrating the contact patterns in Korea^22^ was calibrated with notification of TB incidence data.

### Model parameters

The susceptible individuals move to recently infected (${E}_{S}$) by the force of infection ($\uplambda$), which is proportional to the number of infectious individuals. $1/\nu$ and 1/γ denote the average pre-infectious period and infectious period, respectively. The fraction ($q$) of individuals in $E$ progress to the infectious group ($I$), while the rest progress to the latent group ($L$). Patients in $I$ stay with the unfavorable treatment proportion ($p$), or die from TB with mortality rate (d), derived from TB death reports^23^. If the treatment is successful, infectious individuals progress to long-term latent ($L$) and may transit to ${E}_{L}$ from $L$ by relapse, reinfection, or reactivation with the rate, $\tau$. When people in ${E}_{S}$, ${E}_{L}$, and $L$ are treated, they move to the treatment group ($T$) by the number of preventive therapy ($m$) derived from literature reviews^24^. The force of infection for treated individuals is reduced owing to the partial immunity, and the reduced factor is denoted by $h$^25^. Individuals in the treatment compartment come back to $L$ when they wane, where $1/\rho$ means the duration of waning.

**Figure 3:**
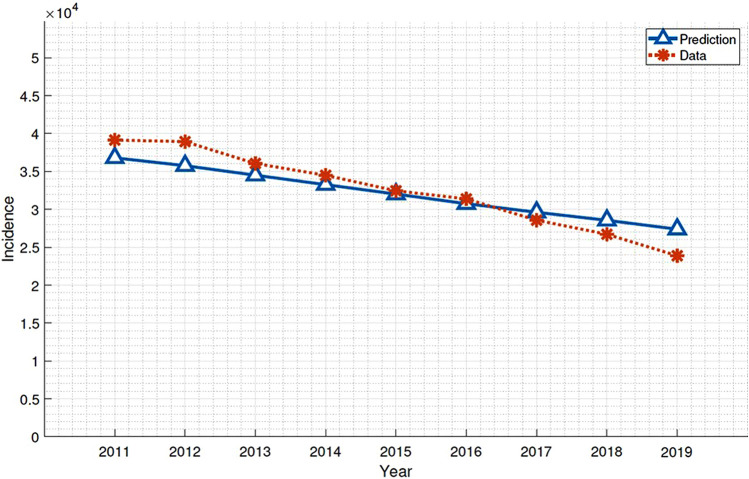
TB incidence by model prediction and the annual TB report data in 2011**–**2019.

Table 1 provides a summary of the description, values of parameters, and references. The values were based on literature reviews, including the annual TB report, investigator's derivations, and mathematical formula. Demographic parameters including population size, birth rate, natural death rate, and aging rate were incorporated from the annual Korea census data. The transmission rate in the force of infection for the age-structured model was represented by Who Acquires Infection From Whom (WAIFW) matrix, which was assumed to be proportional to the contact patterns of Korea^22^. The proportionality factor ($\beta$) and the relapse, reinfection, and reactivation rate ($\tau$) were estimated using the maximum likelihood estimation (MLE) which was calibrated to the number of new patients in the annual TB report data in 2011–2019^19^, whose results are shown in Fig. 3.

**Table 1:** Parameters of TB transmissions model.

Parameter	Description	Values (unit)	References
β	Proportionality factor of transmission rate		Estimated
*q*	Proportion of becoming infectious	5%	^29,30^
1/ν	Pre-infectious period	1.5 (years)	^29,30^
*p*	Unfavorable treatment proportion	6%	Derived^26^
*d*	Mortality rate of tuberculosis		Derived^17^
ℎ	Reduced factor of transmission for preventive therapy	65%	^19^
1/γ	Infectious period	1 (year)	^27,28^
τ	Rate of reactivation, reinfection, or relapse		Estimated
1/ρ	Duration of treatment waning	10 (years)	Assumed
*m*	Number of preventive therapy		Derived^18^

### Scenarios for the impact of COVID-19 on TB

COVID-19 control strategy has variable effects on TB dynamics and management. Health service disruption due to reallocation of resources for COVID-19 care and strong restriction of movement may limit the availability of services. This results in delayed diagnosis and treatment of active and latent TB cases. On the other hand, interventions such as social distancing and wearing a mask have the potential to decrease the transmission of TB by reducing contact among individuals in the community.

We assumed that each effect was represented by changes in the values of the model parameters. Social distancing has been implemented in Korea for a long time, the rule and regulations of which have been adjusted according to the situation, as shown in the diagram (Figure S1). In ongoing research, it has been estimated that one of two different distancing steps, covering most of the duration, reduced transmission by 63% and the other reduced transmission by 50%. In the social distancing scenario, considering a year as a whole, it was assumed that strong distance reduces transmission rate by 63% and moderate distance reduces transmission rate by 50%. For the weak scenario, referring to previous research papers, it was assumed that the beta value decreased by 10%^8^.

Further, we incorporated the level of health service disruption (low, middle, and high) into scenarios expressed as increased treatment failures (9%, 13%, 15%), delayed diagnosis (5%, 10%, 25%), and reduction of the number of close contact management (30%, 50%, 60%). Table 2 summarizes nine scenarios combining social distancing and health service disruption. We assumed that changes in the parameter values due to each scenario lasted for a year during 2020. The cumulative change in TB incidence and deaths by age group over 6 years (2020–2025) were estimated for each scenario and compared to baseline without any change.

**Table 2:** Associated values used for each scenario.

Social distancing		Strong ($\beta$: 63 $\mathrm{\%}\downarrow$)	Moderate ($\beta$: 50% $\downarrow$)	Weak ($\beta$: 10 $\mathrm{\%}\downarrow$)
Health service disruption	Low	$\mathrm{p}=9\mathrm{\%}$	$\mathrm{p}=9\mathrm{\%}$	$\mathrm{p}=9\mathrm{\%}$
		1/$\gamma :5\mathrm{\% delay}$	1/$\gamma :5\mathrm{\% delay}$	1/$\gamma :5\mathrm{\% delay}$
		m: 30% delay	m: 30% delay	m: 30% delay
	Middle	$\mathrm{p}=13\mathrm{\%}$	$\mathrm{p}=13\mathrm{\%}$	$\mathrm{p}=13\mathrm{\%}$
		1/$\gamma :10\mathrm{\% delay}$	1/$\gamma :10\mathrm{\% delay}$	1/$\gamma :10\mathrm{\% delay}$
		m: 50% delay	m: 50% delay	m: 50% delay
	High	$\mathrm{p}=15\mathrm{\%}$	$\mathrm{p}=15\mathrm{\%}$	$\mathrm{p}=15\mathrm{\%}$
		1/$\gamma :25\mathrm{\% delay}$	1/$\gamma :25\mathrm{\% delay}$	1/$\gamma :25\mathrm{\% delay}$
		m: 60% delay	m: 60% delay	m: 60% delay

Summary of scenarios: The effect of social distancing is represented by strong, moderate, and weak levels assuming the reduction of transmission by 63%, 50%, 10%, respectively. The degree of health service disruption is denoted by low, middle, and high considering increase of treatment failures (9%, 13%, 15%), delayed diagnosis (5%, 10%, 25%), and reduction of the number of close contact management (30%, 50%, 60%).

## Results

### Baseline

**Figure 4:**
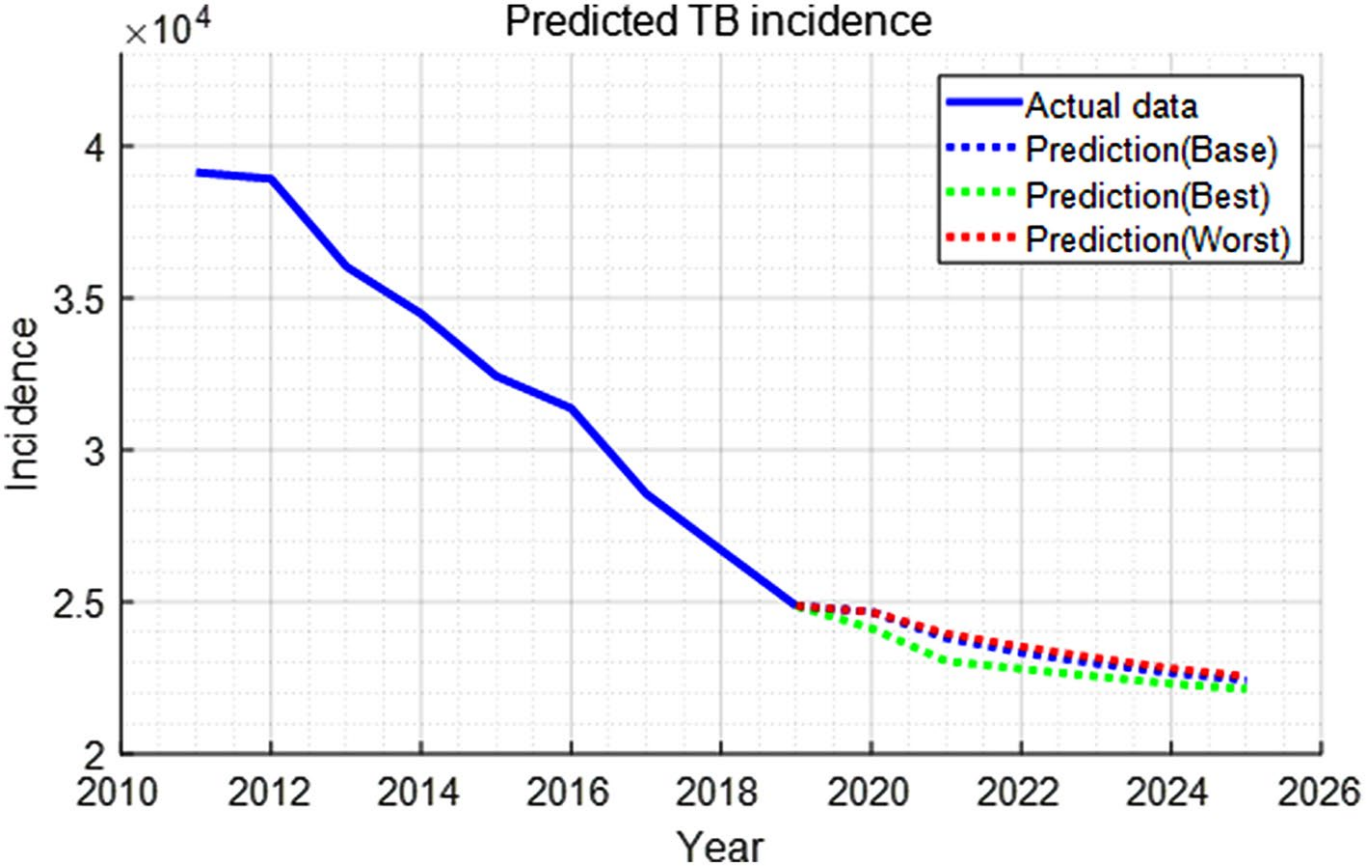
TB incidence data through 2019 and model forecasts for 2020–2025 under different scenarios of social distancing and health service disruption.

**Figure 5:**
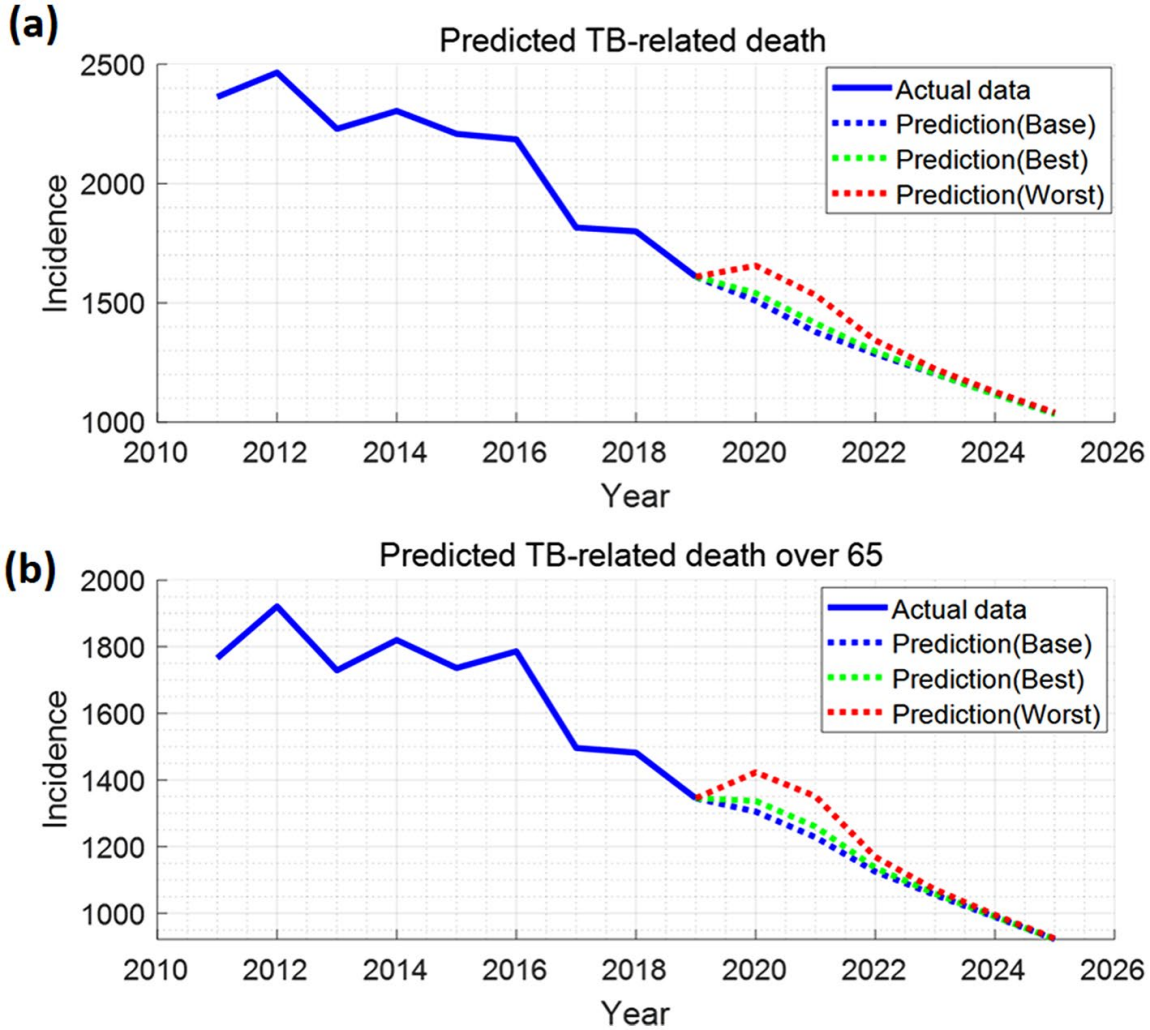
Number of TB-related deaths in all age groups (**a**) and > 65 (**b**): data through 2019 and model forecasts for 2020–2025 under different scenarios of social distancing and health service disruption.

Model simulation predicted that TB incidence and deaths would decrease from 24,680 to 22,427 and from 1509 to 1036, respectively, over a period of next 6 years (Figs. 4, 5, S2, S3). This shows that TB-associated cases and deaths in Korea could continuously decrease if there was no COVID-19 hindering TB management. We noted that TB deaths in people above 65 years of age account for 80%of the total deaths. Hence, changes in this group would strongly affect the overall number of deaths (Figs. 5, S3).

### Impact of COVID-19 on TB

**Figure 6:**
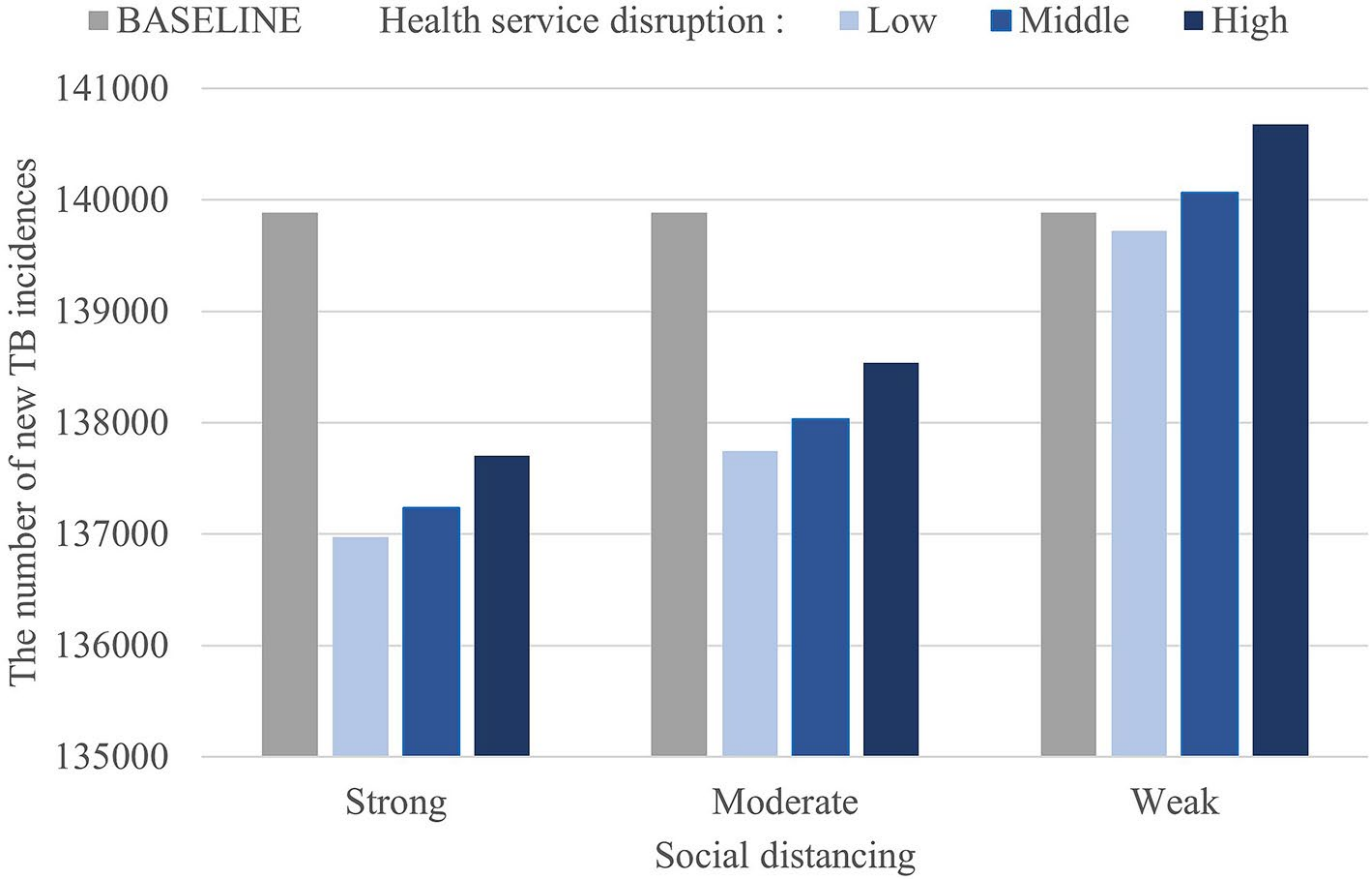
Cumulative TB incidence from 2020–2025 under each scenario of social distancing and health service disruption.

**Figure 7:**
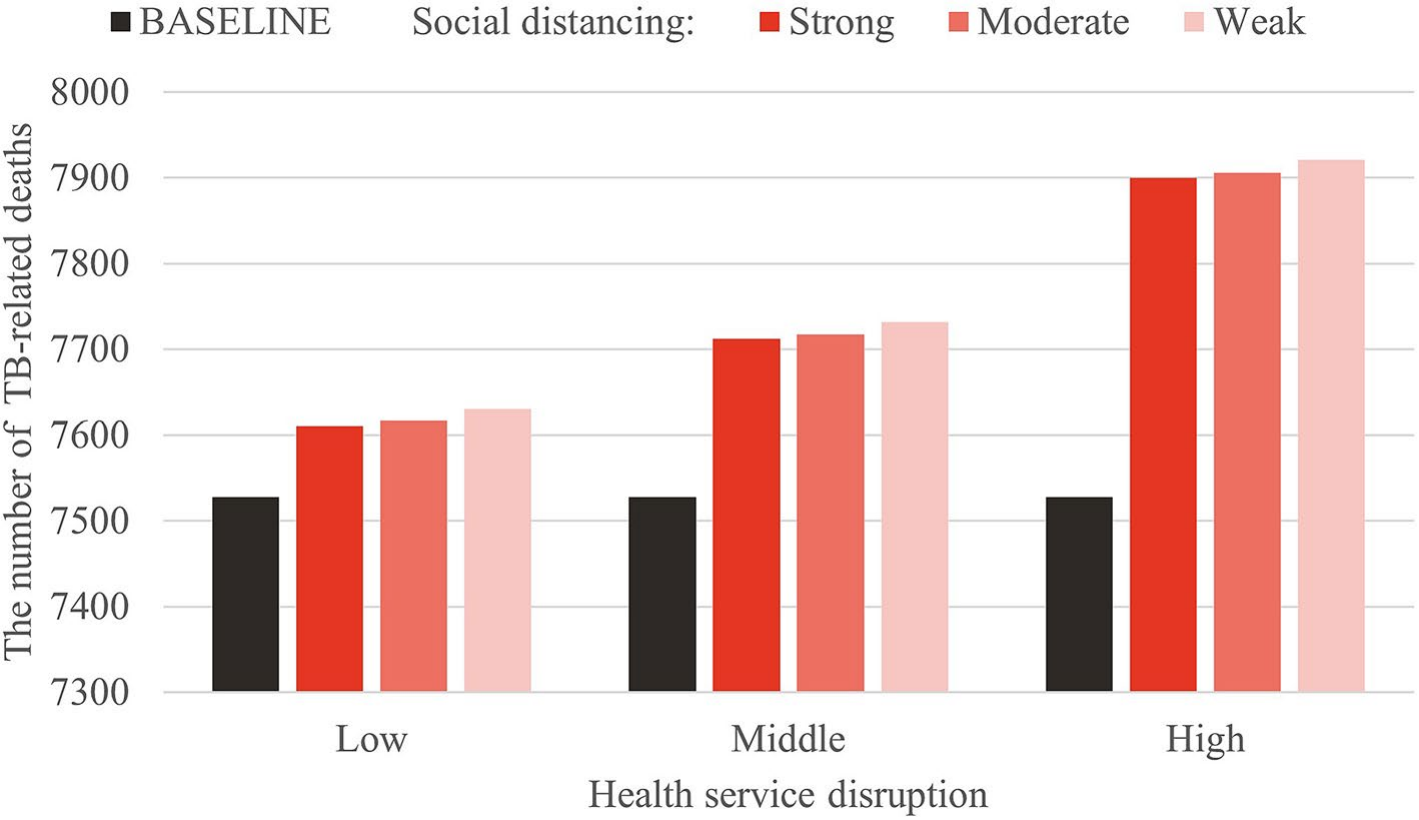
Cumulative number of TB-related deaths from 2020–2025 under each scenario of social distancing and health service disruption.

In each scenario, we calculated the TB incidence and number of deaths for all age groups, and for the > 65 years group, calculations were done for 6 years (2020–2025). Figure 6 shows that the cumulative number of cases decreased with the increase in intensity of social distancing. Under the best scenario of strong social distancing and low-level health service disruption, new TB cases were reduced by 761 after 1 year (Table 3A) compared to the baseline. However, in the elderly population > 65 years old, social distancing had little impact on TB incidence (Table 3B), which illustrated that most active TB cases in the elderly group were either reactivated or relapsed. On the other hand, the number of TB-related deaths mainly depended on the level of health service disruption for TB care. Regardless of the intensity of social distancing, deaths due to TB increased as health services deteriorated (Fig. 7). With a high degree of health service disruption, the number of TB-related deaths would increase up to 155 in 1 year (Table 4A). Moreover, 80% of the TB-related deaths would occur in the elderly population (Table 4B).

**Table 3:** Change in the number of new TB cases under each scenario of social distancing and health service disruptions compared to baseline.

(A) Change in the number of new TB cases of all age groups									
Health service disruption	Social distancing: Strong			Social distancing: Moderate			Social distancing: Weak		
	Low	Middle	High	Low	Middle	High	Low	Middle	High
2020	− 545	− 530	− 511	− 409	− 392	− 367	− 65	− 41	− 3
2021	− 761	− 709	− 621	− 563	− 506	− 407	− 57	16	143
2022	− 535	− 475	− 366	− 390	− 326	− 208	− 18	58	198
2023	− 425	− 372	− 274	− 308	− 252	− 147	− 9	57	179
2024	− 351	− 307	− 224	− 254	− 207	− 119	− 6	48	150
2025	− 294	− 258	− 189	− 213	− 174	− 101	− 6	39	124
Total	− 2911	− 2651	− 2185	− 2138	− 1856	− 1348	− 160	177	791

(B) Change in the number of new TB cases over 65 years of age									
Health service disruption	Social distancing: Strong			Social distancing: Moderate			Social distancing: Weak		
	Low	Middle	High	Low	Middle	High	Low	Middle	High
Total	− 21	− 19	− 18	− 15	− 14	− 12	− 2	0	1

**Table 4:** Change in the number of TB-related deaths under each scenario of social distancing and health service disruptions compared to baseline.

(A) Change in the number of TB-related deaths of all age groups									
Health service disruption	Social distancing: Strong			Social distancing: Moderate			Social distancing: Weak		
	Low	Middle	High	Low	Middle	High	Low	Middle	High
2020	39	39	40	76	77	77	146	146	147
2021	37	38	41	77	78	80	151	152	155
2022	10	11	15	25	26	29	52	54	57
2023	1	2	5	7	8	11	17	18	21
2024	− 2	− 1	2	0	2	4	5	6	9
2025	− 2	− 2	1	− 1	− 1	2	0	1	4
Total	83	88	102	184	189	204	372	378	393

(B) Change in the number of TB-related deaths over 65 years of age									
Health service disruption	Social distancing: Strong			Social distancing: Moderate			Social distancing: Weak		
	Low	Middle	High	Low	Middle	High	Low	Middle	High
2020	32	32	32	62	62	62	118	118	118
2021	32	32	33	64	64	64	122	122	123
2022	11	11	12	23	23	23	44	44	44
2023	4	4	4	8	8	8	15	15	16
2024	1	1	1	3	3	3	5	5	6
2025	0	0	0	1	1	1	2	2	2
Total	81	82	82	160	160	161	306	307	307

## Discussion

Social distancing can help reduce the transmission of TB within the community. However, several studies have shown that TB and COVID-19 have similar symptoms, which results in increased misdiagnosis, thereby affecting family contact transmission^15,18,26^. In addition, TB diagnosis or treatment is delayed owing to limited health service resources, and the period of TB infection is prolonged. Consequently, the TB burden has potentially increased. These consequences are more prominent in low- and middle-income countries due to high TB burden and an incapacitated healthcare system^27,28^.

Several studies suggest that TB mortality and incidence would increase in 2021 and coming years owing to the negative impact of COVID-19^8,15,18,29^. However, it has been predicted that effective social distancing could reduce transmission in countries like India and South Africa, where TB still occurs in young age groups^10^. Research conducted in India, China, and South Africa, which account for two-thirds of the world's total cases, indicate that the impact may vary depending on the epidemiology of TB per country^8,10^.

We simulated nine different scenarios of social distancing and health service disruption to investigate the impact of COVID-19 on TB dynamics. TB incidence and deaths decreased in South Korea despite the presence of COVID-19 response measures in most of the scenarios. However, we found that the pandemic has both positive and negative effects on the TB burden, such as in terms of incidence and deaths. The impact of social distancing was found to be more significant on the number of new cases, which decreased the incidence by reducing the interaction between contacts. On the other hand, the negative effects of health service disruption were observed to be dominant on the increase in number of deaths.

The 2021 WHO TB report showed results similar to that of ours. They reported that the biggest impact of the COVID-19 pandemic on TB was the decrease in TB notifications, which was below the 2019 average^29^. The most immediate effect this change had was the increase in the number of TB-related deaths, which brought the total deaths in 2020 at par with the level reported in 2017^29^.

In this study, the impact of COVID-19 response on TB burden varied with age. Lower TB incidence was evident in the younger age group while higher TB-related deaths were seen among the elderly (Figs. S4, S5). This could be explained by several factors. First, the age-dependent dynamics of transmission and development affect the incidence among age groups differently. Traditionally, TB develops a latent infection which can progress to active infection in the future following exposure and subsequent infection^30^. Historical data suggest that the risk of TB infection is highest during adolescence and young adulthood, while elderly people often develop the disease by reactivation^31^. Young people commonly develop the infectious form of TB, and frequently have a much wider range of social contact outside the household and contribute to the ongoing transmission^32^. Therefore, in regions with high TB prevalence, a substantial burden of TB in young people after community exposure and infection is frequently encountered^33^. In the same context, TB outbreaks in middle and high schools have been reported in Korea^34^. Thus, following COVID-19 social distancing protocols could reduce the incidence and transmission of TB, especially in the younger age groups.

Second, differing health-seeking behavior per age group in the context of COVID-19 response also affects the change in TB-related deaths. TB-related deaths occur mainly in the elderly, which is significantly affected by the health support system. Old age is a known risk factor of severe illness and death from COVID-19, and elderly populations are the major target groups for social distancing and COVID-19 vaccination policies. In this situation, health- seeking behavior may appear differently depending on the age. Older population could be more isolated and reduce their health-seeking behavior. Thus, disruptions in essential health care services could be disproportionately severe. It suggests that maintaining essential health care system during the pandemic is important to control the mortality rate, including TB-related deaths in Korea, where the burden of TB among the elderly is comparatively high.

This study has several strengths. First, we developed a dynamic compartmental mathematical model that could obtain results for age-specific impacts of COVID-19 on TB. Although it is not explicitly revealed in the results, the ages are classified to construct a model that describes the phenomenon better. Through this model, we could implement the various scenarios of social distancing and health service disruption to provide an estimate of TB incidence and number of deaths per age group. Second, the TB transmission model was designed through a set of values based on real data and fitting processes. The reduction in contacts due to social distancing was approximated by analyzing the actual COVID-19 data and contact pattern survey during 2020.

Despite these strengths, this study has some limitations. While the social distancing policy decreases contacts in the community, it may increase family contacts, which was not considered in the study. Second, the degree of health service disruption, such as increased treatment failures, delayed diagnosis, and reduced number of close contact management, were taken into account on the basis of existing literature, which may affect the study results.

## Conclusion

COVID-19 pandemic has variable influences on TB incidence and the number of deaths. The impact of social distancing is dominant on TB incidence, which decreases owing to the reduction of contacts. Moreover, increase of TB-related deaths is significantly affected by health service disruption, which occurs mainly in the elderly.

## Supplementary Information

Supplementary Information.

## Data Availability

The datasets used and/or analyzed during the current study are available from the corresponding author on reasonable request.
