# Supplementary material for: Heterogeneous impact of Covid-19 response on tuberculosis burden by age group
Source: Sci Rep. 2022 Aug 12;12:13773. doi: 10.1038/s41598-022-18135-6 (PMC9374296; doi:10.1038/s41598-022-18135-6)
Supplement: Supplementary file 1 — Supplementary Information. [file 41598_2022_18135_MOESM1_ESM.docx]

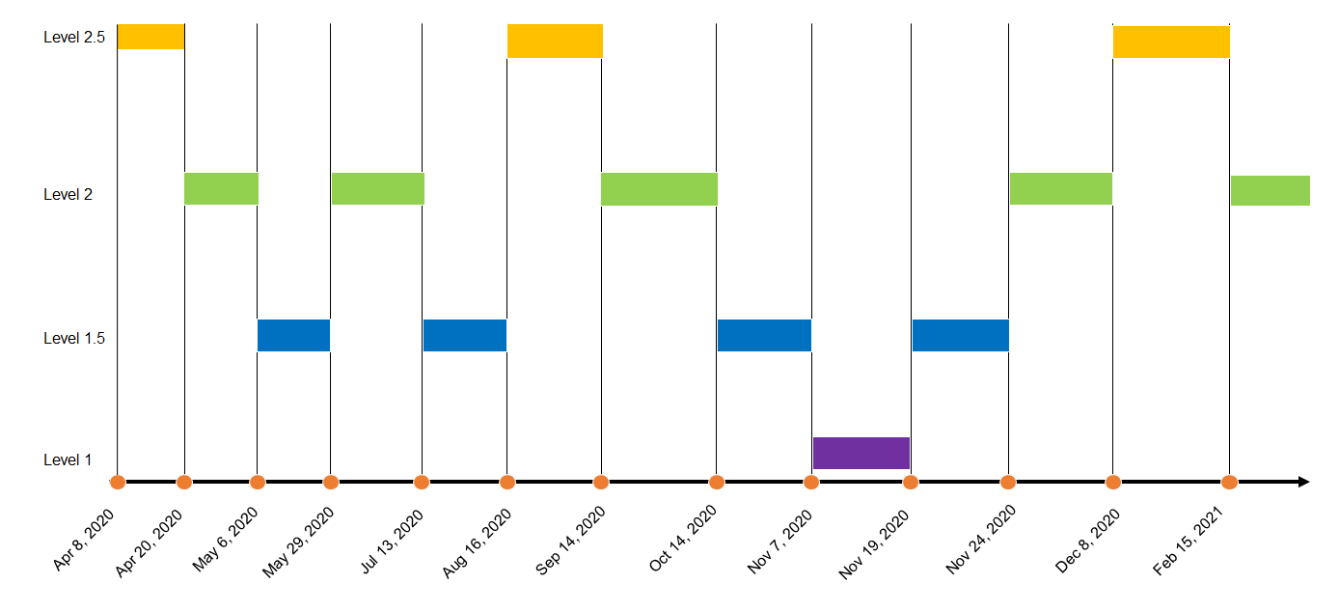


FigureS1. Diagram of social distancing policy change in Seoul metropolitan area from April 8, 2020 to March 11, 2021


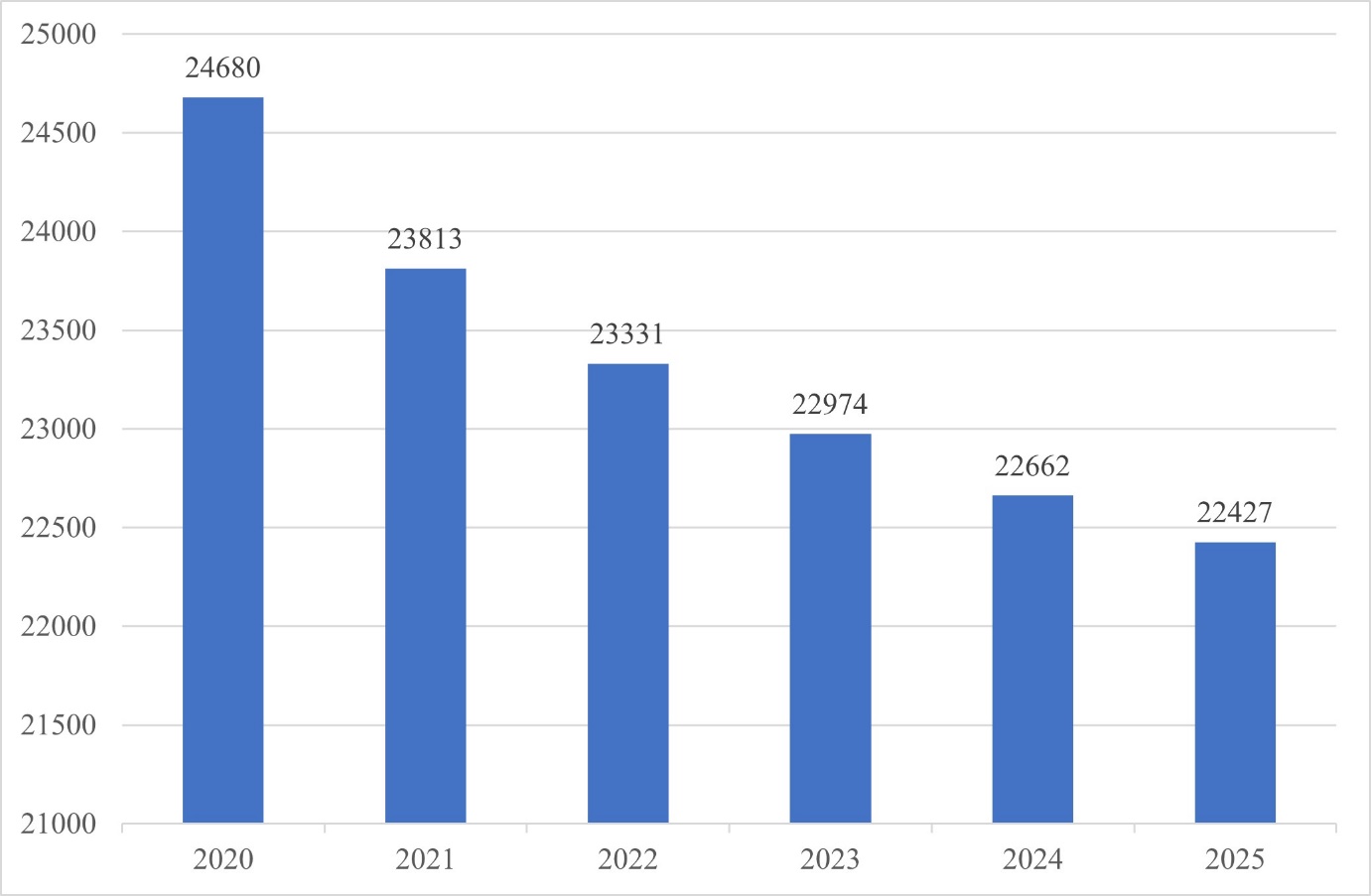


Figure S2. Predicted TB incidence at baseline from 2020–2025 maintaining current situation


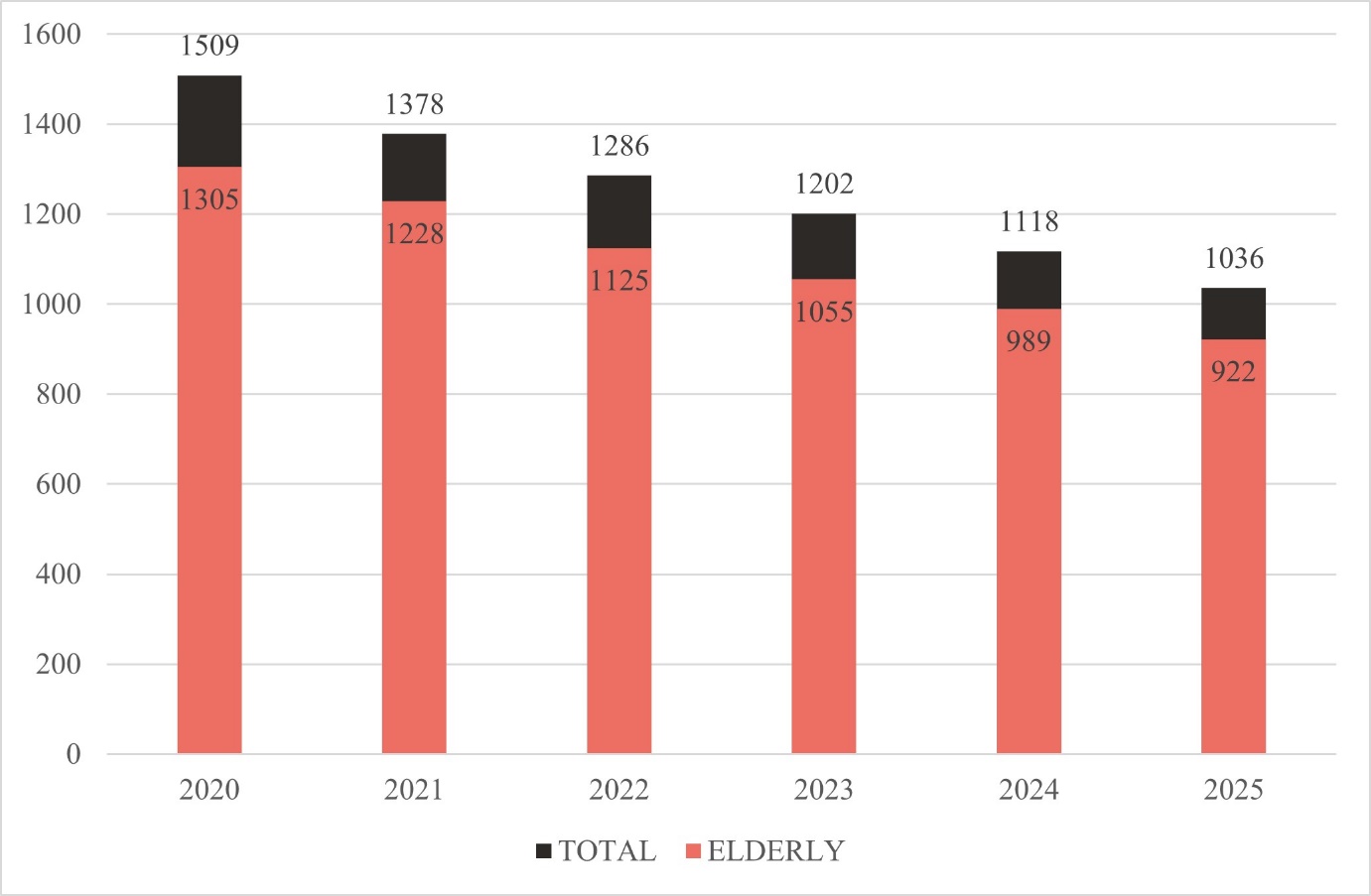


Figure S3. Number of TB-related deaths in all age groups(black) and > 65(red) in baseline from 2020–2025 maintaining the current situation


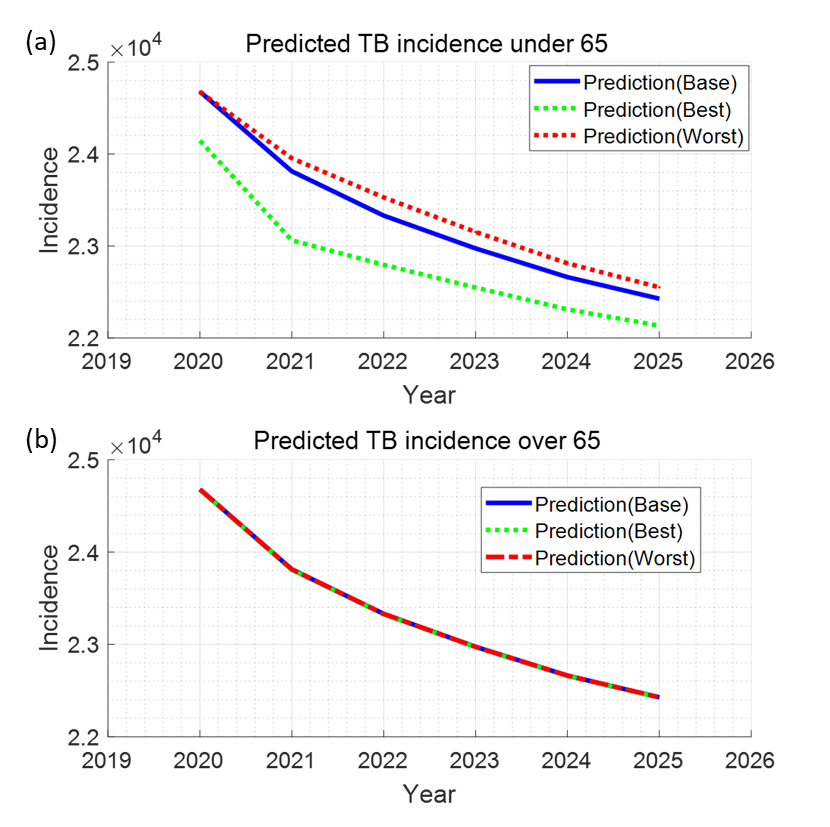


Figure S4. Number of new TB cases for each scenario of social distancing and health care

interruption compared to baseline in the under 65 (a) and over 65 (b) age groups


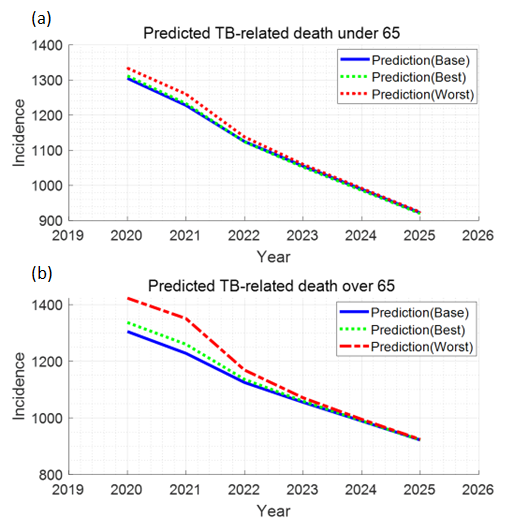


Figure S5. Number of TB-related death for each scenario of social distancing and health care interruption compared to baseline in the under 65 (a) and over 65 (b) age groups
